# Fish Scales as a Non-Invasive Method for Monitoring Trace and Macroelement Pollution

**DOI:** 10.3390/biology14040344

**Published:** 2025-03-26

**Authors:** Haithem Aib, Herta Czédli, Edina Baranyai, Zsófi Sajtos, Boglárka Döncző, Md. Sohel Parvez, Csaba Berta, Zsolt Varga, Ramzi Benhizia, Krisztián Nyeste

**Affiliations:** 1 Pál Juhász-Nagy Doctoral School of Biology and Environmental Sciences, University of Debrecen, 4032 Debrecen, Hungary; haithem.aib@eng.unideb.hu (H.A.); sohel@nstu.edu.bd (M.S.P.); 2 Department of Hydrobiology, University of Debrecen, 4032 Debrecen, Hungary; berta.csaba@science.unideb.hu; 3 Department of Civil Engineering, University of Debrecen, 4028 Debrecen, Hungary; herta.czedli@eng.unideb.hu (H.C.); vzs@eng.unideb.hu (Z.V.); 4 Department of Inorganic and Analytical Chemistry, University of Debrecen, 4032 Debrecen, Hungary; baranyai.edina@science.unideb.hu (E.B.); sajtos.zsofi@science.unideb.hu (Z.S.); 5 HUN-REN Institute for Nuclear Research (ATOMKI), 4032 Debrecen, Hungary; donczo.boglarka@atomki.hu; 6 Department of Oceanography, Noakhali Science and Technology University, Noakhali 3814, Bangladesh; 7 National Laboratory for Water Science and Water Safety, University of Debrecen, 4032 Debrecen, Hungary; 8 Department of Landscape Protection and Environmental Geography, University of Debrecen, 4032 Debrecen, Hungary; benhizia.ramzi@science.unideb.hu

**Keywords:** chub, nase, XRF, ICP-OES, SEM, pollution

## Abstract

Increasing pollution of aquatic ecosystems due to anthropogenic activities underscores the urgent need for effective water quality monitoring. This study evaluates the use of fish scales from chub (*Squalius cephalus*) and nase (*Chondrostoma nasus*) as non-invasive bioindicators of trace and macroelement pollution in the Maros/Mureș River. We conducted qualitative and quantitative elemental analyses using X-ray fluorescence (XRF) and inductively coupled plasma optical emission spectrometry (ICP-OES). Scanning electron microscopy (SEM) revealed distinct scale structures among the species studied. Concentrations of Ca, K, Mg, Na, P, S, Al, Ba, Cu, Cr, Fe, Mn, Sr, and Zn were measured by ICP-OES. Our findings indicate significant variability in elemental accumulation in fish scales, supporting their potential use as bioindicators of environmental pollution, with variability depending on the ecological characteristics of the species. This methodology offers promising applications for modern interdisciplinary monitoring programs.

## Simple Summary

Monitoring the health of aquatic ecosystems is crucial due to increasing pollution from industrial and urban activities. This study explores the use of fish scales as a non-invasive method to monitor trace and macroelement pollution in rivers. By analyzing the scales of two fish species, chub (*Squalius cephalus*) and nase (*Chondrostoma nasus*), from the Maros River, we identified significant differences in elemental accumulation patterns. Advanced techniques such as X-ray fluorescence (XRF) and inductively coupled plasma optical emission spectrometry (ICP-OES) were employed to detect macro- and trace elements. These findings highlight the potential of fish scales as effective bioindicators for environmental pollution while minimizing harm to aquatic organisms. This approach could support sustainable monitoring programs and provide valuable insights into pollution dynamics in freshwater ecosystems.

## 1. Introduction

In the past decades, it has been proven that the spread of trace elements with toxic properties in water bodies can be associated with increasing industrial activity and urbanization [1]. Trace element pollution in aquatic environments is critically important because it can cause irreversible damage to ecosystems and pose significant risks to human health if these pollutants enter the food chain [2,3]. Increasing trace element pollution has significant toxic health effects on invertebrates, fish, and humans due to bioaccumulation and biomagnification The most hazardous trace elements that negatively impact the health and stability of aquatic ecosystems include Ni, Zn, Pb, Cr, Cu, Hg, As, Cd, etc. Environmental scientists need to employ and develop effective analytical methods to assess and monitor concentrations of potentially toxic trace elements in aquatic organisms [4,5]. Water quality has usually been assessed using different physical and chemical techniques. Biomonitoring has been proven to be a necessary supplement to these monitoring techniques. There are different water quality assessment methods for the characterization and management of river systems: single-factor assessment method; water quality grading method; Nemerow pollution index; comprehensive pollution index; principle component analysis; Fuzzy comprehensive evaluation method. However, developing relatively rapid and cost-effective non-invasive methods for monitoring environmental stress and pollution is essential to minimize adverse effects on species and ecosystems [6].

Literature data demonstrate that researchers worldwide have conducted numerous ecotoxicological studies and water monitoring programs on the accumulation of trace and macroelements in various fish tissues [7,8,9,10]. Despite extensive analytical assessments of element accumulation in fish tissues and organs, using fish scales in monitoring programs has not received sufficient attention [11]. Given the need to plan monitoring programs in protected water areas and river sections, where contact with endangered species may occur, lethal sampling is often impractical. Developing non-lethal methods is crucial to prevent and mitigate negative impacts, thereby reducing the number of fish harmed [11]. Additionally, non-lethal methods are advantageous because they allow for repeated analyses and parallel measurements without capturing and killing additional fish for each This approach can obtain samples without capturing and killing additional fish for each measurement. Literature data suggest that the elemental signature of scales remains unchanged after maturation and migration [12]. Elemental signatures in scales from fish in rivers with varying pollution levels can be used for comparative analysis. Studies have shown that scale growth can be arrested during the fish’s life cycle due to various physiological stress factors [13,14]. Scales can regenerate rapidly and change their elemental composition [15]. In environmental analytical studies, scales can serve as environmental tracers to analyze short- and long-term metal and trace element contamination [16].

The calcareous structures of scales and their location in the skin expose them constantly to waterborne pollutants. During the fish’s life cycle, scales grow in size during intensive and less intensive growth periods, incorporating nutrients and pollutants entering the fish’s body. These elements and pollutants are permanently stored in the scales. By determining the age of the fish and analyzing the growth rings on the scales, we can map the pollutants to which the fish was exposed at various stages of its life in the given watercourse [16,17,18]. Using an available and highly effective X-ray analytical method (PIXE), a “toxic period” can be mapped in a given fish by analyzing its scale growth rings [19,20]. In all cases, trace element concentrations in fish scales show a linear correlation with ambient water concentrations [21]. Literature data indicate that metal accumulation in scales can be detected and measured within days of exposure [22]. The main advantage of using scales over other fish tissues is their ability to reveal past pollution and allow chronological ordering of measured values [23,24]. Contact with trace elements can cause ultrastructural damage to fish scales, making them an effective, non-invasive indicator of water pollution during monitoring studies [25,26].

Different trace elements can accumulate to varying extents in fish tissues [27]. The extent of accumulation in different fish species depends on several factors: the physical and chemical properties of the water, the form of the trace element present, the body weight, age, physiological status, and position in the food web [28,29,30]. The aim of the fish scale analysis was to test the efficiency of the two analytical methods used and the development of analytical methods for the analysis of fish scales. In view of the large sample numbers to be collected during field surveys and the cost of analytical analyses, it is necessary to apply a preliminary micro-XRF analytical test to provide qualitative results and to identify, based on the resulting elemental maps, which samples should be analyzed with ICP-OES to obtain quantitative results. These two methods have been chosen for their applicability, testing their efficiency, and evaluating detectable elements.

This study analyzed concentrations of (Al, Ba, Cu, Cr, Fe, Mn, Sr, Zn) and macroelements (Ca, K, Mg, Na, P, S) in the scales of two riverine fish species collected from the River Maros: chub [*Squalius cephalus* (Linnaeus, 1758) previously *Leuciscus cephalus*] and nase [*Chondrostoma nasus* (Linnaeus, 1758)]. These elements are common components of agricultural, urban, and industrial wastewater [31,32]. The River Maros was chosen as the research site due to the significant industrial infrastructure along its banks and in the catchment area, including chemical, pharmaceutical, food, and automotive industries. Our research hypothesized that the studied fish species would exhibit different element levels and bioaccumulation capacities in their scales.

## 2. Materials and Methods

### 2.1. Ethical Approval

The Workplace Animal Experiments Committee of Debrecen University approved the experimental protocol and the end-points of the experiments. All methods followed relevant national and international guidelines and regulations (permission number: HaGF/68/2021). This study complies with the Animal Research: Reporting of in Vivo Experiments (ARRIVE) guidelines.

### 2.2. Sample Collection

The Maros River, one of the largest tributaries of the Tisza River, spans 789 km in Eastern Europe, with a drainage basin covering 30,332 km^2^. It originates in the Hășmașu Mare Range in the Eastern Carpathian Mountains, Romania, and joins the Tisza River at Szeged in southeastern Hungary. In December 2022, we collected eight chub and six nase specimens from the Maros River at Makó using an electric fishing device (Hans Grassl EL64 II GI, DC, 300/600V max. 7 kW, Hans Grassl GmbH, Schönau am Königssee, Germany; permission number: HaGF/68/2021). The geocoordinates of the sampling sites are N46.20276, E20.45203. The fish were transported to the laboratory in containers filled with aerated river water. Each specimen’s standard length (SL) and total weight (W) were measured to the nearest 0.1 mm and 0.01 g, respectively. Following the measurements, the fish were immediately sacrificed by spinal severance to obtain additional biological samples required for comprehensive analysis. While this study utilized fish sacrifice, using scales as non-invasive bioindicators demonstrates the potential for future applications where fish remain unharmed, provided non-lethal sampling techniques are implemented effectively. Scales were collected, washed with distilled water, and stored at −18 °C until sample processing. Based on established methods, scale samples should be collected from six scales on either side of an imaginary line extending from the posterior base of the dorsal fin to the anterior base of the anal fin. Additionally, scales should be taken from within two to three scale rows above the lateral line [33]. All procedures adhered to relevant laws and institutional guidelines and were approved by the appropriate institutional committees (permission number: HaGF/68/2021). The trace element concentration data for water were obtained from the National Environmental Information System of Hungary (OKIR). For this study, the values were taken for 12 months during 2022.

### 2.3. Analytical Measurements ICP-OES

Approximately 0.2 g of the samples were measured on an analytical balance (Precisa ES 225SM-DR), equating to 25 scales per fish, then digested in atmospheric wet digestion by the mixture of 5.0 mL 65% (m/m) HNO_3_ (reagent grade, Merck, Darmstadt, Hesse, Germany) and 1.0 mL 30% (m/m) H_2_O_2_ (reagent grade, Merck). Digested samples were transferred without loss into volume-calibrated plastic centrifuge tubes and diluted up to 15.00 mL with ultrapure water (Synergy UV, MilliporeSigma, Burlington, MA, USA). Solutions were kept at room temperature until further elemental analysis. The elemental concentration of the samples was determined by inductively coupled plasma optical emission spectrometry (ICP-OES 5110 Vertical Dual View, Agilent Technologies, Santa Clara, CA, USA). An autosampler (Agilent SPS4), Meinhard^®^ type nebulizer, and double pass spray chamber were used, and a five-point calibration procedure was applied (ICP VI, Merck, Darmstadt, Hesse, Germany). Standard solutions of the macroelements (Ca, K, Mg, Na, P, S) were prepared from the mono-element spectroscopic standard of 1000 mg L^−1^ (Scharlau, Sentmenat, Barcelona, Spain) and the elements (Ag, Al, B, Ba, Bi, Cd, Co, Cu, Cr, Fe, Li, Mn, Na, Ni, Pb, Sr, Zn) from the multi-element spectroscopic standard solution of 1000 mg L^−1^ (ICP IV, Merck, Darmstadt, Hesse, Germany). In both cases, a 5-point calibration process was used for which standard solutions were diluted with 0.1 M HNO_3_ prepared in ultrapure water. The following wavelength lines of the MP-AES (Microwave Plasma-Atomic Emission Spectroscopy) analysis were used: Ca 317.933 nm, K 766.491 nm, Mg 285.213 nm, Na 589.592 nm, Cd 214.439 nm, Cr 267.716 nm, Cu 324.754 nm, Fe 238.204 nm, Mn 257.610 nm, Pb 220.353 nm, Sr 407.771 nm, and Zn 213.857 nm.

### 2.4. Analytical Measurements XRF

A Bruker M4 TORNADO micro-XRF (Bruker, Billerica, MA, USA) was used for the investigation operating with a Rh X-ray tube at 50 kV accelerating voltage and 200 µA current. On each scale, approximately 1.2 mm × 1 mm area was selected. The spot size was focused to 20 µm by the built-in polycapillary lens. The measurement was performed in air with a velocity of x ms per pixel and y step size. Two energy-dispersive detectors recorded characteristic X-ray lines with a 30 mm^2^ active area. The M-Quant built-in software (version 1.6.621.0) of the M4 TORNADO evaluated the measurement result. For the qualification, the fundamental parameter (FP) approach was applied.

### 2.5. SEM (Scanning Electron Microscope)

The imaging principle of a scanning electron microscope (SEM) is to image the surface of a sample not simultaneously but point-by-point, with a beam of electrons focused on a tiny area. The electrons interact with the material of the sample, and the various signals produced are detected to provide information about the topography and composition of the sample. We aimed to determine the surface differences between the two species via high-resolution electron imaging. This analysis is crucial because the morphology of fish scales can influence their ability to accumulate and retain trace and macroelements. Structural variations, such as scale thickness, surface roughness, and porosity, may impact the interaction between scales and environmental pollutants, providing insights into their suitability for monitoring contamination. After cleaning, the scales were dried in ascending order from ethanol. Based on literature recommendations, in order to avoid ripples, the scale, after the addition of 70% ethanol, was kept between two microscope slides for 2–3 days [34]. The scales were always dried on filter paper [35].

The measurements were carried out in the Laboratory for Heritage Science of the HUN-REN ATOMKI with a type of Jeol, JSM-IT500HR (Jeol, Tokyo, Japan). The scanning electron microscope is suitable for imaging with secondary electrons, backscattered electrons, cathodoluminescence detectors, Raman detectors, and elemental composition with EDS detectors. The SEM measurements and imaging were performed at 20 kV accelerating voltage with 0.05 nA probe current in low vacuum mode, which enables the investigation of non-conductive samples via an adjustable nitrogen atmosphere.

### 2.6. Statistical Analysis

All statistical analyses were performed using the R version 4.4.0. To ensure the appropriateness of statistical methods, the Shapiro–Wilk test was applied to assess the normality of the data. In contrast, Levene’s test was used to evaluate the homogeneity of variances. Due to violations of normality or heterogeneity of variances, non-parametric tests were employed for comparisons.

The Kruskal–Wallis test was conducted to compare macro- and trace element concentrations in the scales between the two species, with post hoc pairwise comparisons performed using the Mann–Whitney U test. Welch’s test was applied to analyze differences in the mean body weights of chub and nase due to unequal variances. The Kolmogorov–Smirnov test was used to compare the weight distributions of the two species.

A Principal Component Analysis (PCA) was performed on trace element concentrations (mg kg^−1^, wet weight) in scales to explore multivariate patterns. The PCA results included 95% confidence ellipses around the points for each species, illustrating the variability and distribution of trace element concentrations within each group.

The Spearman correlation analysis also investigated the relationships between trophic levels and trace element concentrations in fish scales, highlighting varied relationships among the elements.

A significance level of *p* < 0.05 was set for all analyses. Results are presented as test statistics (e.g., H (Kruskal–Wallis test), W (Mann–Whitney U test), or D (Kolmogorov–Smirnov test)) with associated *p*-values.

## 3. Results

### 3.1. Biological Features of Fish

A total of 14 fish (8 chub and 6 nase) were investigated. The mean body weight and standard deviation of chub and nase were 118.88 ± 91.62 g and 59.65 ± 44.79 g, respectively. Statistical analysis indicated no significant difference between the mean body weights of chub and nase (Welch’s *t*-test, t = 1.59, df = 10.66, *p* = 0.1405). However, the observed variability in body weights suggests differences in age among the sampled fish. Since age influences the accumulation of trace elements, this variability may have introduced a confounding factor in comparing contamination levels. Additionally, the weight distributions of the two species did not significantly differ (Kolmogorov–Smirnov test, D = 0.417, *p* = 0.4602). The trophic levels for chub and nase were 2.0 and 2.7, respectively. These values were obtained from the FishBase database.

### 3.2. Concentrations of Elements in Mureș River Water

The descriptive statistics of the concentrations of trace elements in water, obtained from the database of the National Environmental Information System of Hungary (OKIR in Hungarian), are presented in Table 1. Only the mean concentrations of Fe were above the criterion chronic concentrations (CCCs) for the freshwater of the National Recommended Water Quality Criteria prescribed by the USEPA.

### 3.3. Analysis of Macro- and Microelements

This study evaluated the macro elements (Ca, K, Na, Mg, P, S) and trace elements (Ag, Al, B, Ba, Bi, d, Co, Cr, Cu, Fe, Li, Mn, Ni, Pb, Sr, Zn) for their various physiological and bioaccumulation features in scales. The mean concentrations of macro- and trace elements are summarized in Table 2. The Kruskal–Wallis test was conducted to compare the concentration levels of different elements in the scales of chub and nase.

For macroelements, significant differences were observed only for S, where the test indicated a notable difference between the two species (H = 8.07, df = 1, *p* < 0.005). In contrast, no significant differences were found for Ca (H = 1.67, df = 1, *p* = 0.197), K (H = 0.15, df = 1, *p* = 0.699), Na (H = 3.27, df = 1, *p* = 0.071), Mg (H = 2.82, df = 1, *p* = 0.093), or P (H = 3.75, df = 1, *p* = 0.053) (Table 2).

For trace elements, the concentrations of Ag, B, Bi, Cd, Co, Li, Ni, and Pb were below the detection limit, indicating that these elements were not present at measurable levels in the samples. The Kruskal–Wallis test identified significant differences between species for Ba (H = 8.07, df = 1, *p* < 0.01), Fe (H = 3.75, df = 1, *p* < 0.05), Mn (H = 6.67, df = 1, *p* < 0.01), and Sr (H = 4.82, df = 1, *p* < 0.05). No significant differences were found for Al (H = 0.82, df = 1, *p* = 0.366), Cr (H = 3.05, df = 1, p = 0.081), Cu (H = 0.02, df = 1, *p* = 0.897), or Zn (H = 1.21, df = 1, *p* = 0.272) (Table 2).

**Figure 1:**
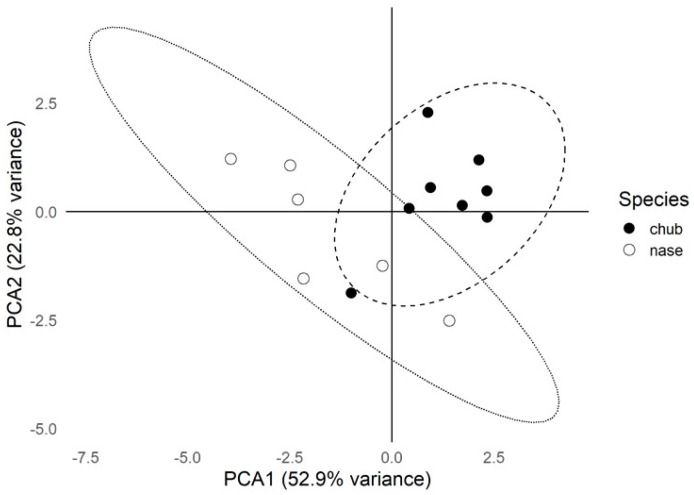
Principal Component Analysis (PCA) of trace element concentrations (mg kg^−1^, wet weight) in the scales of chub and nase from the Maros River. The circles around the points represent each species’ 95% confidence ellipses, indicating the variability and distribution of trace element concentrations within each group.

The PCA revealed distinct separations between chub and nase based on their trace element concentrations in scales. The first principal component (PCA1) accounted for 52.9% of the total variance, indicating that it captures a substantial portion of the variability in the data. The second principal component (PCA2) contributed 22.8% of the total variance, highlighting additional patterns of variation between the species (Figure 1).

### 3.4. Correlation of Element Concentrations with Trophic Level and Fish Weight

The Spearman correlation analysis between trophic levels and trace element concentrations in fish scales revealed varied relationships among the elements. A significant positive correlation was observed for sulfur (S), with a correlation coefficient of 0.79 (*p* < 0.001), suggesting potential biomagnification in this case. Significant negative correlations were found for phosphorus (P) (correlation coefficient: −0.54, *p* < 0.05), barium (Ba) (correlation coefficient: −0.79, *p* < 0.001), iron (Fe) (correlation coefficient: −0.54, *p* = 0.05), manganese (Mn) (correlation coefficient: −0.72, *p* < 0.01), and strontium (Sr) (correlation coefficient: −0.61, *p* = 0.05) (Table 3).

The remaining elements, including calcium (Ca), potassium (K), magnesium (Mg), sodium (Na), aluminum (Al), and copper (Cu), did not show significant correlations with trophic levels (Table 3).

Spearman correlation analysis revealed several significant associations between scale trace element concentrations and fish weight for both species. For chub, weight showed significant positive correlations with sodium (Na, *p* < 0.001) and phosphorus (P, *p* < 0.05), while a significant negative correlation was observed with zinc (Zn, *p* < 0.01). For nase, weight was significantly negatively correlated with aluminum (Al, *p* < 0.05), chromium (Cr, *p* < 0.01), copper (Cu, *p* < 0.05), and iron (Fe, *p* < 0.05). Other elements did not show significant correlations with weight for either species (Table 4).

### 3.5. XRF Analysis

In the case of fish scales, preliminary analysis is particularly important to select samples and speed up laboratory work, allowing qualitative analyses to be carried out more cost-effectively. Based on the XRF scales analysis (Table 5; these results are not normalized to 100%), it can be concluded that the method used is well suited for the preliminary examination of fish scales for selection analysis and arbitrarily selected elements, the resulting elemental maps can be used to compare the fish scales by qualitative analysis. It is a fast, efficient method, does not require lengthy sample preparation, is cost-effective, and provides visually appealing results. In the case of larger sample sizes, pre-selection measurement is recommended to select those samples with high confidence of higher concentrations of the elements of interest. The present study focused exclusively on elements detectable in fish scales.

### 3.6. SEM Analysis

**Figure 2:**
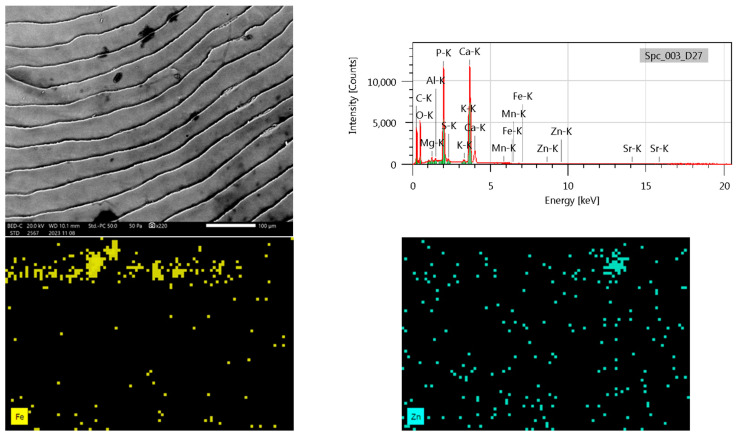
Scanning electron microscopy image with X-ray fluorescence spectra and elemental mapping of iron and zinc in chub scale samples.

**Figure 3:**
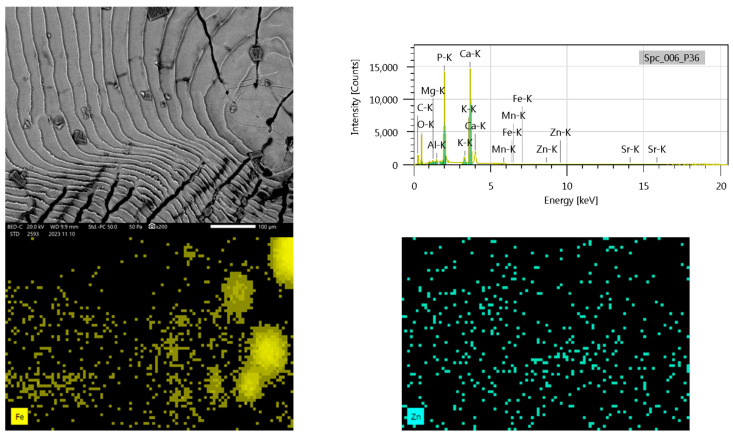
Scanning electron microscopy image with X-ray fluorescence spectra and elemental mapping of iron and zinc in nase scale samples.

We aimed to determine the surface differences between the two species using high-resolution electron imaging. Scanning electron microscopes (SEMs) use a focused electron beam to scan a selected sample and produce images of the samples’ topography and composition. Microstructural components of fish scales considered to be of taxonomic importance are focus, chromatophores, rays, and circles. Some of these parameters are prominent in different fish groups, in others, they are less pronounced. During the SEM analysis of the Chub and Nase scales, we confirmed the morphological differences in the scales (Figure 2 and Figure 3).

We have identified typical ordered architectural patterns on the surface of the basal part of the scale; we plan to analyze the differences between the investigated species during our further investigations. In the present study, on the SEM of the scales, in some cases, we observed a difference between the two species scale element parameters and scales between the surface architecture of its basic parts.

Scale morphology is the result of different genetic and environmental influences. Scale chemistry reveals biogeochemical properties and is a bioindicator of habitat contamination. Literature data show that the elemental surface area of fish scales is closely related to the aquatic environment in which the fish grew up and lived permanently.

During the elemental mapping of the surface of the fish scales, we showed the presence of P, S, Cl, K, Ca, Mg, Fe, Ni, Zn, and Sr. Since fish scale samples are heterogeneous, the elemental composition may differ in specific areas of the scale depending on the position of the SEM images. The element maps are not absolute concentration maps, but only X-ray intensity maps in all cases. The method(s) used during our measurements were micro-XRF (Bruker M4 TORNADO) and Jeol SEM.

In the case of chub, the elemental composition analysis revealed distinct spectral patterns. The peak mass and atomic percentage of carbon indicate its dominance in the spectral line. Oxygen exhibits discrepancies between different spectral configurations. Potassium fluctuates in mass percent and atomic composition across spectral configurations. Calcium (Ca) and phosphorus (P) display variations in both mass% and atomic%, following similar patterns. The sulfur (S) content is inconsistent, while the mass% and atomic% of potassium (K) vary between spectral lines, highlighting the importance of considering spectral patterns. Manganese (Mn) is present in all configurations, whereas strontium (Sr) and zinc (Zn) show variable concentrations (Figure 2).

In the case of nase, the heavy metal composition of nase scales exhibited varying patterns for manganese (Mn), iron (Fe), and strontium (Sr). Manganese was below the detection limit in most scales, with only minor concentrations in some samples. Iron levels were consistently low across all scales, with several values below the detection limit, and the highest concentrations found in two scales. Strontium concentrations were generally low, with only one scale showing a noticeably higher concentration. These findings emphasize the heterogeneity of the elemental composition of nase scales across different sampling sites, particularly regarding Mn, Fe, and Sr content (Figure 3).

## 4. Discussion

This study used fish scales from the Maros River, specifically those of chub and nase, as non-invasive bioindicators for tracking trace and macroelement pollution. Previous studies have also demonstrated the effectiveness of fish scales as bioindicators [32,33]. For instance, research on common carp scales has shown their ability to reflect environmental heavy metal concentrations [36]. Similarly, Coello and Khan [37] found that fish scales can accumulate metals earlier than other tissues, corroborating our results showing that elements such as Ba, Mn, Sr, and Zn were present in varying concentrations in the scales. This suggests that fish scales are a first line of defense against trace element pollution [37]. The results revealed notable differences in elemental concentrations between the two species, supporting the idea that fish scales reflect species-specific ecological traits and environmental conditions. Although elements absorbed in the fish scales could be sourced from both the environment as well as from their diets [38]. However, the concentrations of macro- and microelements in the examined samples are likely to have been influenced by environmental factors. While the scales serve as tools for analysis, the chub and nase act as bioindicators due to their ability to reflect environmental conditions through elemental accumulation. Unlike previous research that predominantly relied on lethal sampling techniques targeting tissues such as muscle or liver, this study highlights the advantages of using scales as non-lethal alternatives. Given that fish scales preserve elemental signatures throughout the fish’s life, existing studies have demonstrated their efficacy in capturing trace element variability over time [6,15]. Our findings align with these observations [31]. This study did not conduct a direct analysis correlating metal concentrations in water and scales. However, prior studies have established that scales can reflect environmental metal concentrations under specific conditions [16]. To strengthen these findings, future research could focus on quantitative correlations between water chemistry and scale composition. This study builds upon prior research by employing advanced analytical methods such as ICP-OES and XRF, which, together, provide robust qualitative and quantitative assessments. ICP-OES offers superior sensitivity and broader elemental coverage, making it ideal for trace-level analysis and a wide range of sample types. The XRF is a versatile technique suited for qualitative and semi-quantitative analysis, especially when analyzing samples with minimal preparation. When both analysis methods are used, maximum accuracy is achieved. When the required limits of quantification are above 1 ppm (µg/g) or when non-destructive analysis is required, XRF is a very good technique that should be considered, especially for fish scales. The XRF method used is well suited for the preliminary examination of fish scales for selection analysis and arbitrarily selected elements. The resulting elemental maps can be used to compare the fish scales by qualitative analysis. The use of scanning electron microscopes (SEM) offers a wide range of applications in the analysis of fish scales.

The observed variations in elemental bioaccumulation between chub and nase scales may be attributed to their physiological characteristics, preferred habitats, and trophic positions. Chub (2.0) and nase (2.7) trophic levels were obtained from the FishBase database. These trophic positions provide insights into the ecological variability that influences elemental bioaccumulation. For example, higher levels of manganese (Mn) and barium (Ba) in nase scales may reflect dietary intake specific to this species or localized pollution sources within its habitat. These findings are consistent with Wright’s discussion on trophic-level influences on bioaccumulation [31].

Although fish scales effectively retain elemental signatures, their utility for local contamination monitoring is limited by fish mobility. This movement allows for the accumulation of contaminants from multiple environments, which may obscure localized pollution signals. Since the nase moves over a longer section of a given river due to migration, it can be considered an indicator species for pollutants in the stretch of the river channel it moves during migration. Additionally, the potential for scales to be lost and regrown introduces variability in elemental concentrations, necessitating a consistent collection of scales from the exact anatomical location for comparative analyses.

The multivariate analysis further revealed distinct pollution profiles between species, emphasizing the importance of considering interspecies ecological variability when interpreting bioindicator data. Species-specific variations in elemental accumulation may be linked to differences in ecological roles and feeding behaviors between chub and nase. Prior research has established that feeding patterns and habitat preferences influence heavy metal accumulation among fish species [38,39]. The observed differences in elemental accumulation between chub and nase align with findings from Jordanova [40], which reported variations in heavy metal uptake linked to species-specific feeding behaviors and habitat preferences. For example, nase exhibited higher Mn and Ba levels, likely due to localized dietary sources and environmental exposure.

This research underscores the potential of fish scales as non-invasive tools for environmental monitoring, offering a means to reconstruct historical pollution timelines and assess the long-term impacts of human activities while aligning with ethical practices in sensitive ecosystems [41].

The relatively small sample size (eight chub and six nase) is a limitation of this study and may restrict the generalizability of the findings. Future research should include larger sample sizes across broader spatial and temporal scales to validate the observed patterns of elemental accumulation. The significant relationships between fish weights and trace elements suggest that biological factors such as growth rates and age may influence metal accumulation [40,42]. The observed variability in elemental concentrations across scales likely reflects differences in fish age, growth rates, and environmental exposures. Age-related factors, such as metabolic activity and duration of exposure to pollutants, may contribute to the differences in bioaccumulation. Standardizing the age or size of sampled fish could help mitigate this variability in future studies. These findings are consistent with studies indicating that younger fish may exhibit distinct accumulation patterns compared to older individuals due to differences in exposure duration and metabolic rates [34].

This study focused on various macroelements and trace elements commonly found in the Maros River. While toxic metals like Pb and Hg are critical for ecotoxicological assessments, their concentrations were below detection limits in the sampled scales, possibly due to the specific industrial activities in the study area.

Despite these promising results, some limitations of this study must be acknowledged. The relatively small sample size and the need for a more comprehensive spatial and temporal dataset should be addressed in future research. Expanding the scope to include diverse aquatic environments while integrating sediment and water quality assessments with fish scale data could provide a more holistic understanding of pollution dynamics. Fish scales offer significant advantages as non-lethal indicators of environmental contamination. Their ability to retain elemental signatures over time makes them valuable for reconstructing historical pollution events [43]. However, limitations such as interspecies variability, fish mobility, and the potential for scale regrowth must be considered when interpreting results. Advances in analytical techniques, such as synchrotron radiation and PIXE, could enhance the precision of future biomonitoring efforts.

In this study, only trace elements were included in the PCA analysis because these elements are typically associated with anthropogenic pollution sources and exhibit significant variability across different environmental gradients. Conversely, macroelements are more uniformly distributed in aquatic environments and are less indicative of localized contamination patterns. Therefore, focusing on trace elements allowed us better to explore pollution-related differences between species and sampling sites.

One notable advantage of using fish scales as indicators of metal contamination is their ability to serve as archival materials for assessing past contamination levels. However, the accuracy of XRF analysis can vary depending on factors such as element type, sample characteristics (e.g., shape and homogeneity), concentration levels, and accompanying elements. Quantitative results from XRF analysis can also assess potential hazards associated with consuming contaminated fish.

## 5. Conclusions

During this research, we examined the elemental content of fish scales, revealing significant variations and patterns of bioaccumulation. The XRF method proved an effective preliminary analytical tool for analyzing fish scales. Examining fish scales is crucial in elemental analytical research as it provides insights into the life history of fish, bioaccumulation processes, the entry pathways of various chemical elements into the fish’s body, and the water quality of aquatic environments. Research involving different fish species highlights their role as bioindicators for monitoring changes in water quality and identifying the routes through which heavy metals enter aquatic ecosystems. The importance of fish scales as biomarkers deserves special attention in hydrobiological research due to their ability to absorb heavy metal impurities from aqueous solutions.

The findings emphasize the critical need for monitoring and managing river heavy metal pollution. The preliminary elemental analysis of fish scales conducted using the XRF method demonstrated that this technique is effective for detecting various heavy metals and determining their radial distribution within the scales. Analytical analysis using the ICP method provided a comprehensive view of element concentrations in fish scales.

This study demonstrates that XRF analysis is an effective and reliable technique for examining micro and macro elements in fish scales and other environmental samples. Fish scales act as potential non-lethal water quality indicators and provide valuable insights into pollution dynamics within aquatic ecosystems.

## Figures and Tables

**Table 1:** Descriptive statistics of total trace element concentrations in water from the Maros River obtained from the National Environmental Information System of Hungary (OKIR in Hungarian) database for 2022.

	Trace Element Concentration (μg L^−1^) in Water			
Element	Min	Max	Mean ± SD	Threshold Values (μg L^−1^) ^a^
Al	1.05	13	4.68 ± 3.68	–
Cd	BDL	BDL	BDL	0.72
Cr	0.005	0.042	0.016 ± 0.011	74
Cu	0.008	0.051	0.019 ± 0.012	3.1
Fe	729	12,500 *	4725 ± 3769 *	1000
Mn	48	446	176 ± 138	–
Ni	0.006	0.073	0.026 ± 0.027	52
Pb	0	0.013	0.00475 ± 0.00411	2.5
Zn	0.006	0.084	0.033 ± 0.025	120

^a^ Criterion chronic concentrations (CCCs) for the freshwater of National Recommended Water Quality Criteria (EPA, 2018). * The concentration of trace elements in the water was higher than the threshold value.

**Table 2:** Mean concentrations (±standard deviation) of macro- and trace elements in scales of chub and nase from the Maros River (mean value ± SD).

(A) Trace Element Concentrations in Scales		
Element	Chub	Nase
Ca	95,445 ± 14,151	106,815 ± 13,627
K	3388 ± 758	3205 ± 651
Mg	1771 ± 234	2222 ± 516
Na	1194 ± 760	254 ± 570
P	36,810 ± 6724	44,160 ± 8207
S	3486 ± 466	3135 ± 86
Al	19.10 ± 9.62	21.60 ± 7.83
Ba	15.90 ± 11.20 *	53.70 ± 14.80 *
Cr	1.19 ± 0.24	1.49 ± 0.31
Cu	1.19 ± 0.23	1.37 ± 0.83
Fe	3.29 ± 0.83 *	8.39 ± 5.19 *
Mn	12.63 ± 18.29 *	24.15 ± 15.81 *
Sr	143.88 ± 34.72 *	175.63 ± 23.23 *
Zn	116.31 ± 25.07	124.93 ± 35.96

* The values in the same row are significantly different (Kruskal–Wallis test, *p* < 0.05).

**Table 3:** Correlation coefficients between element concentrations in scales and species’ trophic levels in the Maros River; significant at *p* < 0.05 (*N* = 14).

Elements	
Ca	n.s.
K	n.s.
Mg	n.s.
Na	n.s.
P	−0.537
S	0.788
Al	n.s.
Ba	−0.788
Cr	n.s.
Cu	n.s.
Fe	−0.537
Mn	−0.716
Sr	−0.608
Zn	n.s.

n.s.: non-significant.

**Table 4:** Spearman correlation coefficients between element concentrations in scales and fish weight for chub and nase from the Maros River (significance at *p* < 0.05; *N* = 8 for chub, *N* = 6 for nase).

Elements	Chub	Nase
Ca	n.s.	n.s.
K	n.s.	n.s.
Mg	n.s.	n.s.
Na	0.976	n.s.
P	0.738	n.s.
S	n.s.	n.s.
Al	n.s.	−0.886
Ba	n.s.	n.s.
Cr	n.s.	−0.928
Cu	n.s.	−0.943
Fe	n.s.	−0.943
Mn	n.s.	n.s.
Sr	n.s.	n.s.
Zn	−0.905	n.s.

n.s.: Non-significant.

**Table 5:** Elemental concentrations in fish scales of chub and nase across varying body weights. The table presents the net values and weight percentages ([wt.%]) for elements detected using XRF analysis, including P, S, Cl, Ca, Mn, Fe, Ni, Zn, and Sr. Data are grouped by species (chub and nase) and individual fish weights, providing detailed elemental profiles helpful for comparative qualitative analysis.

Chub Nase	27 g 28 g		44 g 45 g		91 g 91 g		136 g 136 g		144 g 214 g	
Elements	Net	[wt.%]	Net	[wt.%]	Net	[wt.%]	Net	[wt.%]	Net	[wt.%]
P	876,642 1,211,275	2.802 3.800	1,038,717 1,103,236	3.268 3.505	1,091,565 1,091,683	3.437 3.400	1,163,305 1,071,610	3.627 3.303	1,088,533 1,157,732	3.367 3.545
S	40,880 33,852	0.066 0.053	55,711 30,680	0.087 0.049	40,357 21,198	0.062 0.032	19,330 29,009	0.029 0.043	38,870 26,708	0.058 0.039
Cl	5291 11,653	0.006 0.012	15,968 18,459	0.017 0.020	5360 8914	0.005 0.009	28,916 23,876	0.029 0.023	13,257 397	0.013 0.000
K	350,902 1,301,317	0.210 0.766	544,726 1,253,031	0.316 0.758	1,000,170 665,354	0.574 0.372	1,284,764 847,532	0.734 0.469	905,771 748,642	0.511 0.405
Ca	32,815,220 49,917,523	15.338 23.660	44,028,122 41,693,283	20.362 20.146	50,029,439 53,381,183	23.222 24.203	54,347,832 53,534,851	25.304 24.137	51,046,833 61,727,312	23.351 27.379
Mn	8714 31,190	0.002 0.008	16,100 83,140	0.004 0.022	95,788 79,092	0.025 0.021	55,151 103,415	0.015 0.027	95,521 122,320	0.025 0.032
Fe	54,899 60,783	0.011 0.012	61,036 21,371	0.013 0.004	14,458 42,687	0.003 0.009	34,542 19,321	0.007 0.004	10,640 26,369	0.002 0.005
Ni	0 0	0.000 0.000	0 0	0.000 0.000	0 0	0.000 0.000	0 0	0.000 0.000	0 0	0.000 0.000
Zn	199,301 251,143	0.023 0.029	220,916 210,903	0.026 0.024	196,200 223,368	0.023 0.026	220,839 433,660	0.026 0.050	250,431 263,751	0.029 0.031
Sr	179,287 231,336	0.019 0.025	195,473 221,365	0.021 0.024	255,344 258,470	0.028 0.028	288,463 276,279	0.032 0.030	272,870 342,732	0.030 0.037

## Data Availability

Dataset available on request from the authors.
